# Angiotensin-Converting Enzyme Inhibitor, Captopril, Improves Scar Healing in Hypertensive Rats

**DOI:** 10.7150/ijms.50197

**Published:** 2021-01-01

**Authors:** Eun Young Rha, Jae Won Kim, Jun Hyeok Kim, Gyeol Yoo

**Affiliations:** Department of Plastic and Reconstructive Surgery, College of Medicine, The Catholic University of Korea, Seoul, Republic of Korea

**Keywords:** angiotensin-converting enzyme inhibitor, Wistar Kyoto rat, spontaneously hypertensive rat, scar area, fibrosis, vascular endothelial growth factor

## Abstract

Pathological cutaneous scars, with aberrant extracellular matrix accumulation, have multiple origins. Antihypertensive medications, such as calcium channel blockers, have been used to treat pathological scars. However, a relationship between angiotensin-converting enzyme (ACE) inhibitors, pathological scars, and blood pressure (BP) has never been reported. Here, we aimed to compare the differences in scar development and the effects of the administration of systemic ACE inhibitor on scar tissue in a normotensive rat, the Wistar Kyoto rat (WKY), a hypertensive rat, and the spontaneously hypertensive rat (SHR). Using an 8-mm punch, we created two full-thickness skin defects in a total of 32 rats (16 WKY and 16 SHR) to obtain a total of 64 wounds. We established control WKY (n = 16), captopril-treated WKY (n = 16), control SHR (n = 16), and captopril-treated SHR (n = 16) groups and started captopril (100 mg/g per day) treatment on day 21 in the appropriate groups. The BP of all groups was measured at 0, 3, and 5 weeks. The scar area was measured by histopathological examination, and scarring was expressed in terms of scar area and fibroblast and capillary counts. The expression of heat shock protein (HSP) 47, type I and III collagens, alpha-smooth muscle actin (α-SMA), Ki67, and vascular endothelial growth factor (VEGF) was investigated using immunohistochemistry. The scar area and fibroblast count were significantly higher in control SHR than in control WKY. The scar area, fibroblast count, and capillary count were significantly smaller in captopril-treated SHR than in control SHR. Immunostaining for α-SMA, Ki67, and VEGF also showed a noticeable decrease in scarring in the treated SHR compared with that in control SHR. Thus, BP affects scar development in a rat model, and an ACE inhibitor is more effective at reducing scars in hypertensive rats than in normotensive rats.

## Introduction

Keloid and hypertrophic scars, which are pathological cutaneous scars, are characterized by prolonged, aberrant extracellular matrix (ECM) accumulation 1. They are considered dermal fibro-proliferative lesions rather than an inevitable postoperative outcome in patients with high aesthetic expectations 2.

The development of pathological scars has multiple etiologies, including single nucleotide polymorphisms, local mechanical tension, systemic metabolic adenosine triphosphate levels, antinuclear antibodies, endocrinological sebum secretion, and nutritional fatty acids 3. Recently, it was hypothesized that hypertension is a factor in pathological scar development 1. Antihypertensive medications such as angiotensin-converting enzyme (ACE) inhibitors and calcium channel blockers have been used to treat pathological scars 4, 5. Clinical and animal studies have described the effect of the systemic administration of ACE inhibitors on pathological scars 4, 6, 7. Uzun et al. reported that early application of enalapril after dermal injury reduces scar formation using rabbit ear hypertrophic scar model 7. Fang et al. 8 demonstrated that ACE inhibitors suppress the TGF-β1/SMAD2/3 and TGF-β1/TAK1 pathways in vivo and in vitro using mouse NIH 3T3 fibroblasts cultured with different concentrations of ACE inhibitors and a rat model of full-thickness skin wound. However, no studies have directly demonstrated a relationship between ACE inhibitors, pathological scars, and blood pressure (BP).

Given this background, we compared the differences in scar development and the effects of the systemic administration of an ACE inhibitor, captopril, on cutaneous scar tissues in Wistar Kyoto rats (WKY), a genetically related normotensive control strain, and spontaneously hypertensive rats (SHR), a genetically related hypertensive strain.

## Materials and Methods

### Experimental Setting and Animals

We used a total of 32 male rats (16 male WKY and 16 male SHR (SLC, Nakaizu, Japan)) at the age of 12-13 weeks, weighing 250-350 g. The animals were kept in individual cages in a temperature-controlled room with a 12 h light/12 h dark cycle and were provided food and water ad libitum. All animal experiments were performed to minimize suffering.

### Experimental Rat Model

The WKY and SHR were anesthetized by an intraperitoneal injection of sodium pentobarbital (40 mg/kg). After shaving the hair on the dorsal surface, two full-thickness excisional wounds were created through the panniculus carnosus on the back using an 8-mm biopsy punch. After hemostasis was achieved by manual compression, the wounds were covered with polyurethane dressing (Tegaderm, 3M Health Care, St. Paul, MN). A total of 64 wounds were examined grossly for signs of infection, desiccation, and epithelialization at 1-day intervals. All wounds were epithelialized on day 14.

### Treatment

The 16 WKY and 16 SHR were randomly assigned into four groups: Control WKY (WKY-C, n = 16), captopril-treated WKY (WKY-T, n = 16), control SHR (SHR-C, n = 16), and captopril-treated SHR (SHR-T, n = 16). Between days 21 and 35 after wounding, the WKY-T and SHR-T groups were treated with captopril (100 mg/kg per day added to drinking water) using a dosage regimen adapted from previous studies 9. The WKY-C and SHR-C groups served as the normal control, receiving water only. The body weight and systolic BP were measured at weeks 0, 3, and 5 using a non-invasive tail-cuff system (IITC Life Science, Woodland Hills, CA).

### Tissue Preparation

A total of 32 rats (8 rats in each group) were euthanized on day 36 after wounding, and a total of 64 tissue biopsies were performed to evaluate inflammatory cell counts and scar areas. Tissue samples (10 mm thick) were collected from the middle region of the wounds. All specimens were fixed in 10% neutral-buffered formalin, dehydrated, embedded in paraffin blocks, cut into 3-µm sections, and stained with hematoxylin and eosin (H&E) and Masson's trichrome stain for examination under a light microscope to measure the scar area, collagen deposition and neovasculature 7, 8, 10.

### Histopathology

The severity of the scarring in the tissue specimens was determined using the scar area, fibroblast count, and capillary count. For histopathological examinations, digital images were captured using Leica Application Suite Software LAS V 4.1 (Leica Microsystems, Wetzlar, Germany). The scar area was defined as the area showing the destruction of the standard architecture compared with the surrounding area, as measured by tracing the scar margin using ImageJ software (ImageJ; National Institute of Health, Bethesda, MD, USA) 11. The mean scar area for each wound was then converted from pixel number to square micrometers, calculated using the ratio of the number of pixels to the scale bar 12. Blind measurements were performed on the scars, and two values were averaged. The number of fibroblasts in three randomly chosen fields of 1 mm^2^ was counted under 400x magnification, and the number of capillary lumens per field was counted under 40x magnification by two histologists blinded to the groups.

### Immunohistochemistry

Immunohistochemistry was performed on formalin-fixed paraffin-embedded (FFPE) 4-μm-thick sections using a Benchmark Ultra autostainer (Roche Tissue Diagnostics, Tucson, AZ, USA) and the UltraView^TM^ universal DAB detection kit (Ventana), according to the manufacturer's instructions. Paraffin sections were immunostained with primary antibodies against HSP47 (diluted 1:300, monoclonal EPR4217; GeneTex, CA, USA), collagen I (diluted 1:100, monoclonal 3G3; Abcam, Cambridge, MA, USA), collagen III (diluted 1:100, polyclonal ab7778; Abcam), Ki67 (clone 30-9, Ventana), actin (clone 1A4, Ventana), and vascular endothelial growth factor (VEGF) (ab1316) (Abcam). The scar tissue was scored using a semi-quantitative approach based on the proportion of positively stained cells to assess HSP47, collagen I, collagen III, and actin immunostaining as follows: (-) absence of staining; (1+) weak staining; (2+) moderate staining; (3+) strong staining. Basal cells (100 cells) were counted in three random locations in a tissue section (100× magnification) to calculate the Ki67 proliferation index, after which the number of positive cells along the length of the epidermis was determined. The proliferation index was defined as the average percentage of Ki67-positive nuclei, as previously described 13. VEGF expression was evaluated in vessels and inflammatory cells (mainly lymphocytes, plasma cells, and neutrophils) and assessed semi-quantitatively based on the sum of the staining intensity (0, negative; 1, weak; 2, moderate; and 3, strong) and the distribution of positive cells in five random scar fields in each sample (0, <10%; 1, 11-50%; 2, >50%). A score exceeding 4 indicated strong VEGF expression; otherwise, it indicated weak expression.

### Ethical Considerations

This study was approved by the Animal Care and Use Committee of Yeouido St. Mary's Hospital, Catholic University of Korea (YEO20141003T).

### Statistical Analysis

Statistical analysis was performed using SPSS ver. 18.0 for Windows (SPSS, Chicago, IL, USA). All the data are expressed as the mean ± standard deviation (SD). The scar area, fibroblast count, and capillary count in all of the groups were compared using one-way analysis of variance (ANOVA). HSP47, type I and III collagen, and VEGF expression scores, as well as the Ki67 proliferation index, were compared using the Kruskal-Wallis test, followed by posthoc Bonferroni correction for multiple comparisons. We also used repeated-measures ANOVA to compare the systolic BP between 3 and 5 weeks in all of the groups. *P*- values < 0.05 were considered statistically significant.

## Results

### Captopril Treatment Reduces Systolic Blood Pressure in Normotensive and Hypertensive Rats (Systolic BP Values Changed at Different Timepoints)

**Figure 1:**
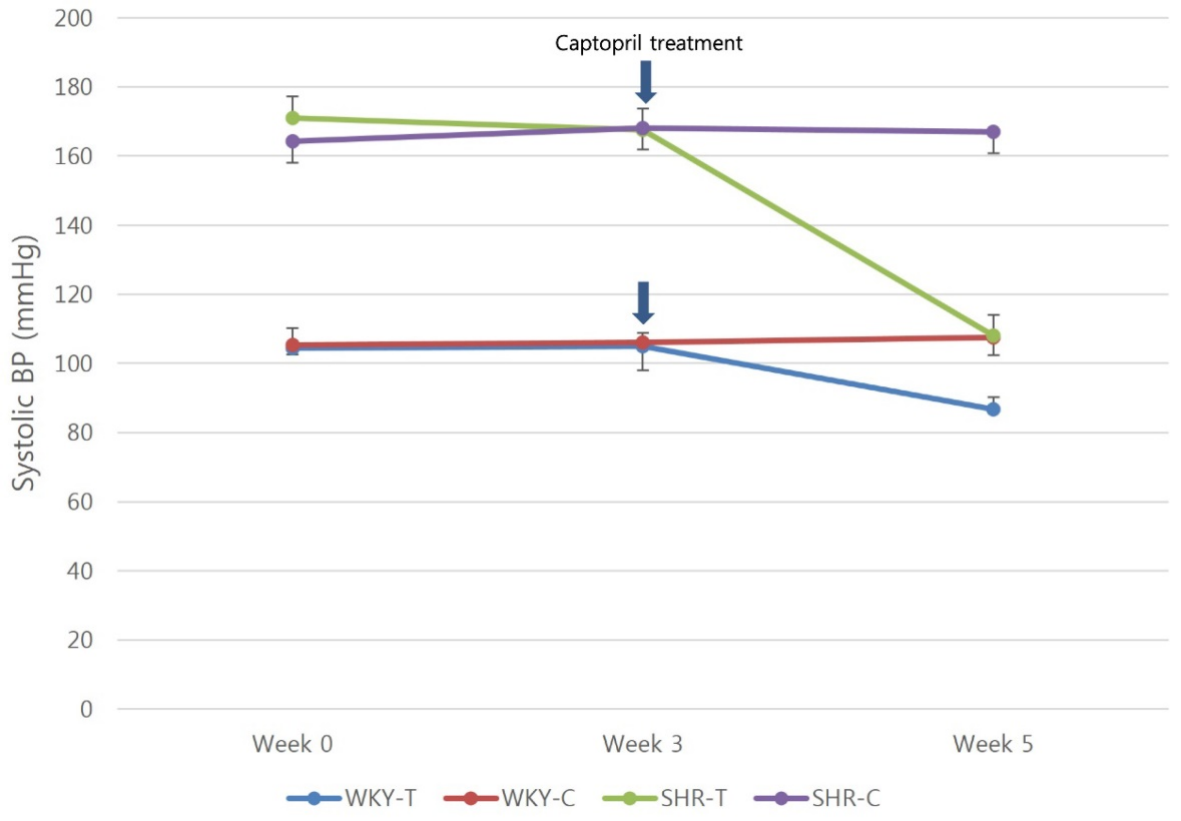
**Captopril treatment reduces systolic blood pressure in normotensive and hypertensive rats.** Serial systolic blood pressure in the Wistar Kyoto rat group (red and blue lines) and spontaneously hypertensive rat group (purple and green lines). Arrows show the starting point of captopril treatment. WKY-C, control Wistar Kyoto rats; WKY-T, captopril-treated Wistar Kyoto rats; SHR-C, control spontaneously hypertensive rats; SHR-T, captopril-treated spontaneously hypertensive rats. Statistical significance was considered at *P* < 0.05 based on repeated-measures ANOVA followed by a *post hoc* Bonferroni correction for multiple comparisons.

At 3 weeks (baseline), the mean systolic BPs of the WKY-C, WKY-T, SHR-C, and SHR-T groups were 106 ± 8, 104.9 ± 3.9, 168.1 ± 6.6, 167.6 ± 6.2 mmHg, respectively; at 5 weeks (2 weeks after treatment), they were 107.6 ± 5.1, 86.8 ± 3.4, 167.1 ± 6.9, 108 ± 6.2 mmHg, respectively. In WKY-T and SHR-T, the mean systolic BP at 5 weeks was significantly lower compared with that at 3 weeks (P < 0.0001) (Figure 1).

### Captopril Treatment Inhibits Scar Area in Hypertensive Rats In Vivo

**Figure 2:**
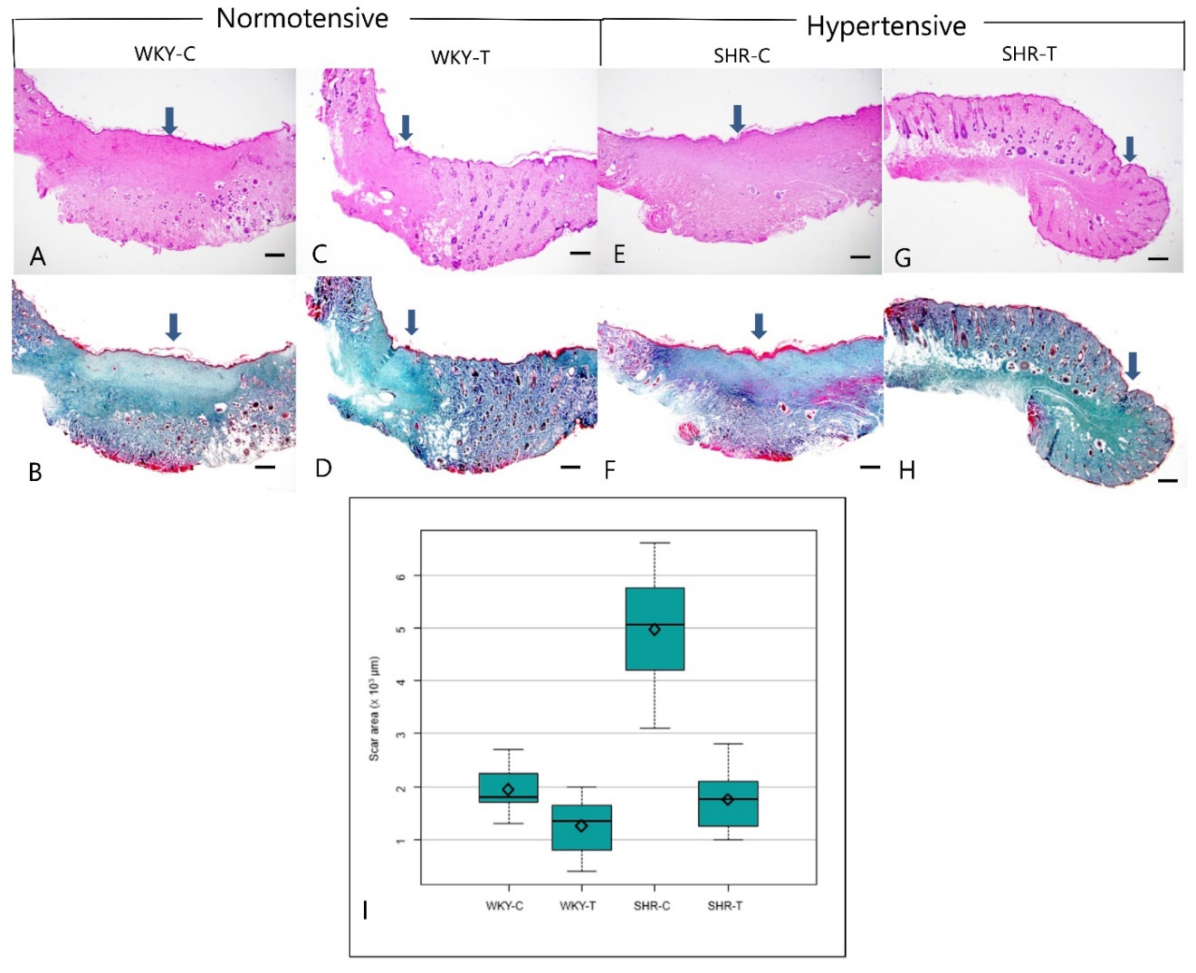
** Histopathological change between normotensive and hypertensive rats after captopril treatment. Captopril treatment significantly inhibits scar area in hypertensive rats.** (A, B) Control Wistar Kyoto rat. A prominent scar area (arrow) includes fibroblast proliferation and dense connective tissue (A, Hematoxylin & eosin, B, Masson trichrome, 20×). (C, D) Captopril-treated Wistar Kyoto rat. The scar area (arrow) is less marked, and collagen is coarsely distributed (C, Hematoxylin & eosin; D, Masson trichrome, 20×). (E, F) Control spontaneously hypertensive rat. A prominent scar (arrow), characterized by fibroblast proliferation, dense connective tissue, and many capillary lumens, occupies much of the total area (E, Hematoxylin & eosin; F, Masson trichrome, 20×). (G, H) Captopril-treated spontaneously hypertensive rat. The scar area (arrow) makes up a smaller part of the total area and contains sparsely distributed collagen (G, Hematoxylin & eosin; H, Masson trichrome stain, 20×). (I) The mean scar area differed significantly among the four groups. Captopril treatment significantly inhibits scar area in hypertensive rats. Scale bars in all images are 500 μm. WKY-C, control Wistar Kyoto rats; WKY-T, captopril-treated Wistar Kyoto rats; SHR-C, control spontaneously hypertensive rats; SHR-T, captopril-treated spontaneously hypertensive rats. *P* < 0.0001 with posthoc Bonferroni correction.

Histopathological analysis revealed larger scar areas, comprising a dense connective tissue, a less organized collagen structure, and many capillary lumens, in the WKY control group than in the captopril-treated WKY group. Compared with the SHR control group, the captopril-treated SHR group showed a focal scar area with less prevalent fibroblasts, loosely arranged collagen bundles, and fewer capillary lumens (Figure 2A-2H). The median scar area differed significantly among all four groups (*P* < 0.0001). In addition, the scar area was significantly smaller in the wounds of SHR-T and WKY-C than in those of SHR-C. However, the differences between the wounds of WKY-C and WKY-T and WKY-T and SHR-T rats were not significant (Figure 2I and Table 1).

### Captopril Treatment Inhibits Fibrosis in Hypertensive Rats In Vivo

**Figure 3:**
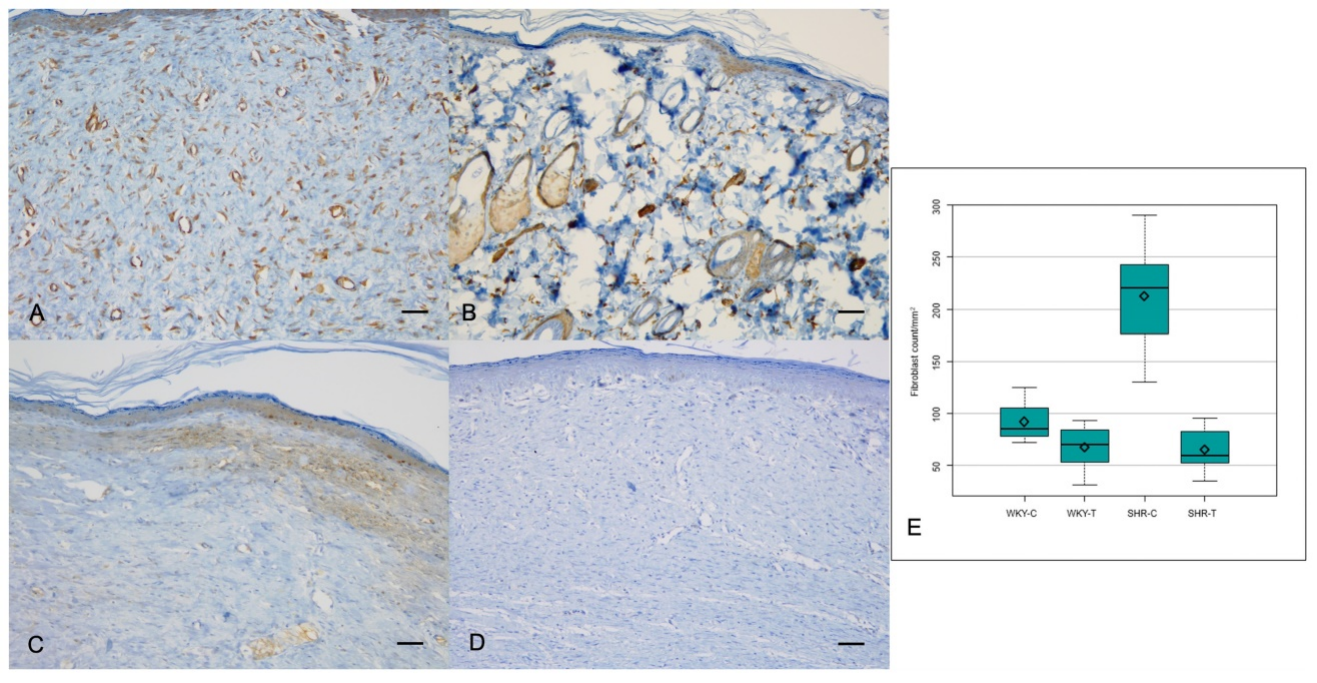
** Immunostaining for α-SMA and Ki67 proliferation index. Captopril treatment inhibits fibrosis in hypertensive rats.** (A) Intense α-SMA staining is seen in the control spontaneously hypertensive rat tissues, and (B) weak staining in the captopril-treated spontaneously hypertensive rat tissues (100×). (C) Strong Ki67 immunoreactivity is seen in the control spontaneously hypertensive rat tissues and (D) weak immunoreactivity in the captopril-treated spontaneously hypertensive rat tissues (100×). (E) The mean fibroblast count differed significantly among the four groups. Scale bars in all images are 50 μm. WKY-C, control Wistar Kyoto rats; WKY-T, captopril-treated Wistar Kyoto rats; SHR-C, control spontaneously hypertensive rats; SHR-T, captopril-treated spontaneously hypertensive rats. *P* < 0.0001 with posthoc Bonferroni correction.

The α-SMA expression, however, showed a significant difference among the four groups (*P* = 0.0000); it was significantly lower in the wounds of WKY-T and SHR-T than in those of WKY-C and SHR-C, respectively (Figure 3A, 3B and Table 2). The Ki67 proliferation index differed significantly among the four groups (*P* < 0.0000) and was significantly lower in the wounds of SHR-T and WKY-C than in those of SHR-C. There were no significant differences in KI67 proliferation index between the wounds of WKY-C and WKY-T or between those of WKY-T and SHR-T (Figure 3C, 3D, and Table 2).

The median fibroblast count differed significantly among all four groups (*P* < 0.0001). The fibroblast counts were significantly lower in the wounds of SHR-T and WKY-C than in those of SHR-C. Furthermore, the difference in fibroblast counts between the wounds of WKY-C and WKY-T and between those of WKY-T and SHR-T was not significant (Figure 3E and Table 1).

### Captopril Treatment Inhibits Scar Deposition by Inhibiting Capillary Count and VEGF Expression in Normotensive and Hypertensive Rats

**Figure 4:**
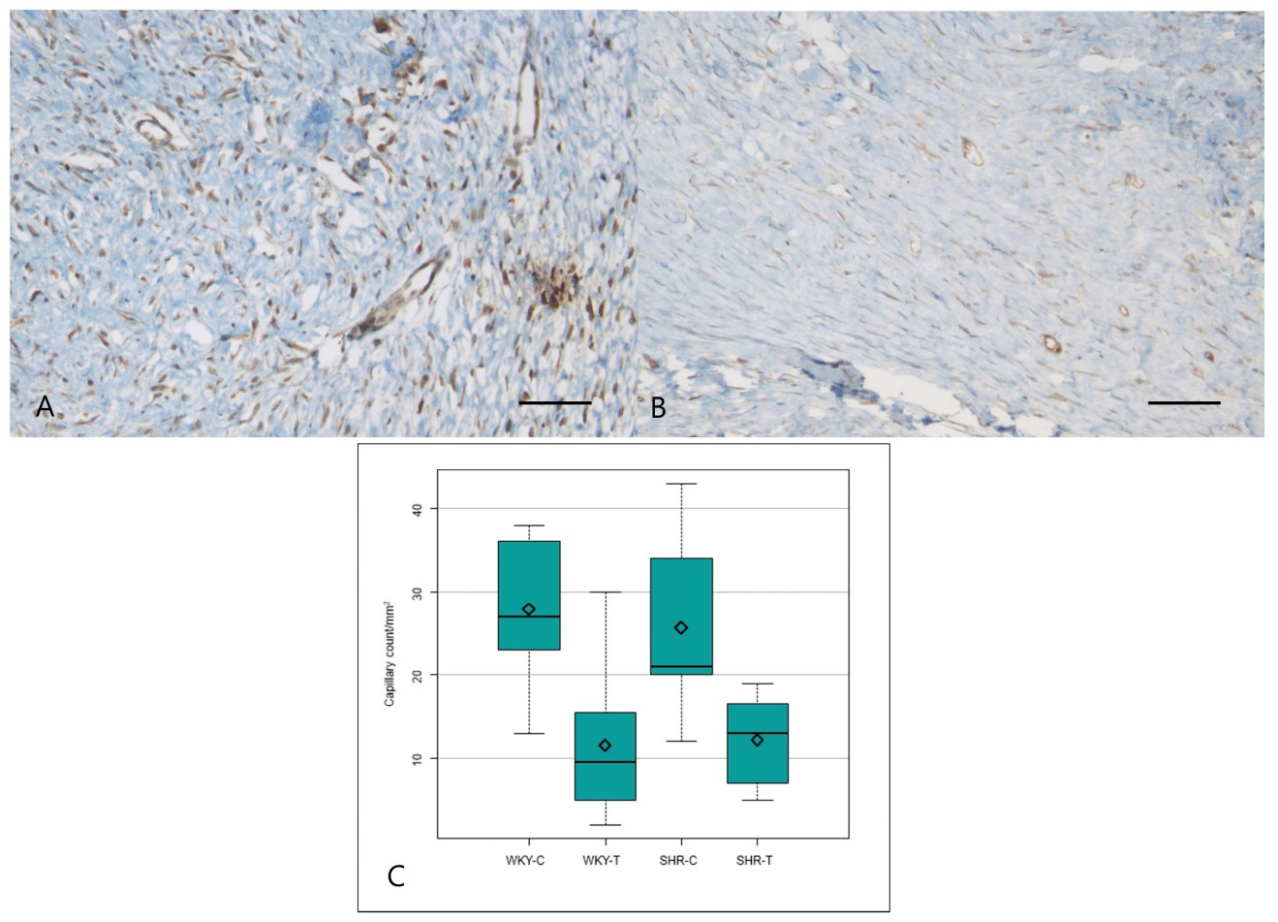
** Immunostaining for VEGF and mean capillary count in each group. Captopril treatment inhibits scar deposition by inhibiting capillary count and VEGF in normotensive and hypertensive rats.** (A) Strong VEGF immunoreactivity is seen in the control spontaneously hypertensive rat tissues and (B) weak VEGF immunoreactivity in the captopril-treated spontaneously hypertensive rat tissues (200×). (C) The mean capillary count differed significantly among the four groups. Scale bars in all images are 50 μm. WKY-C, control Wistar Kyoto rats; WKY-T, captopril-treated Wistar Kyoto rats; SHR-C, control spontaneously hypertensive rats; SHR-T, captopril-treated spontaneously hypertensive rats. *P =* 0.0004 with post hoc Bonferroni correction.

The VEGF expression score differed significantly among the four groups (*P* = 0.000) and was significantly lower in the wounds of WKY-T than those of WKY-C and in the wounds of SHR-T than those of SHR-C. There was no significant difference between the wounds of WKY-C and SHR-C (Figure 4A, 4B and Table 2).

The median capillary count also differed significantly among all four groups (*P* = 0.0004). Capillary counts were significantly lower in the wounds of WKY-T and SHR-T than in those of WKY-C and SHR-C, respectively. The differences between the wounds of WKY-C and SHR-C were not significant (Figure 4C and Table 1).

### Captopril Treatment Has No Effect on Collagen I, Collagen III, and HSP47 Expression in Hypertensive Rats In Vivo

**Figure 5:**
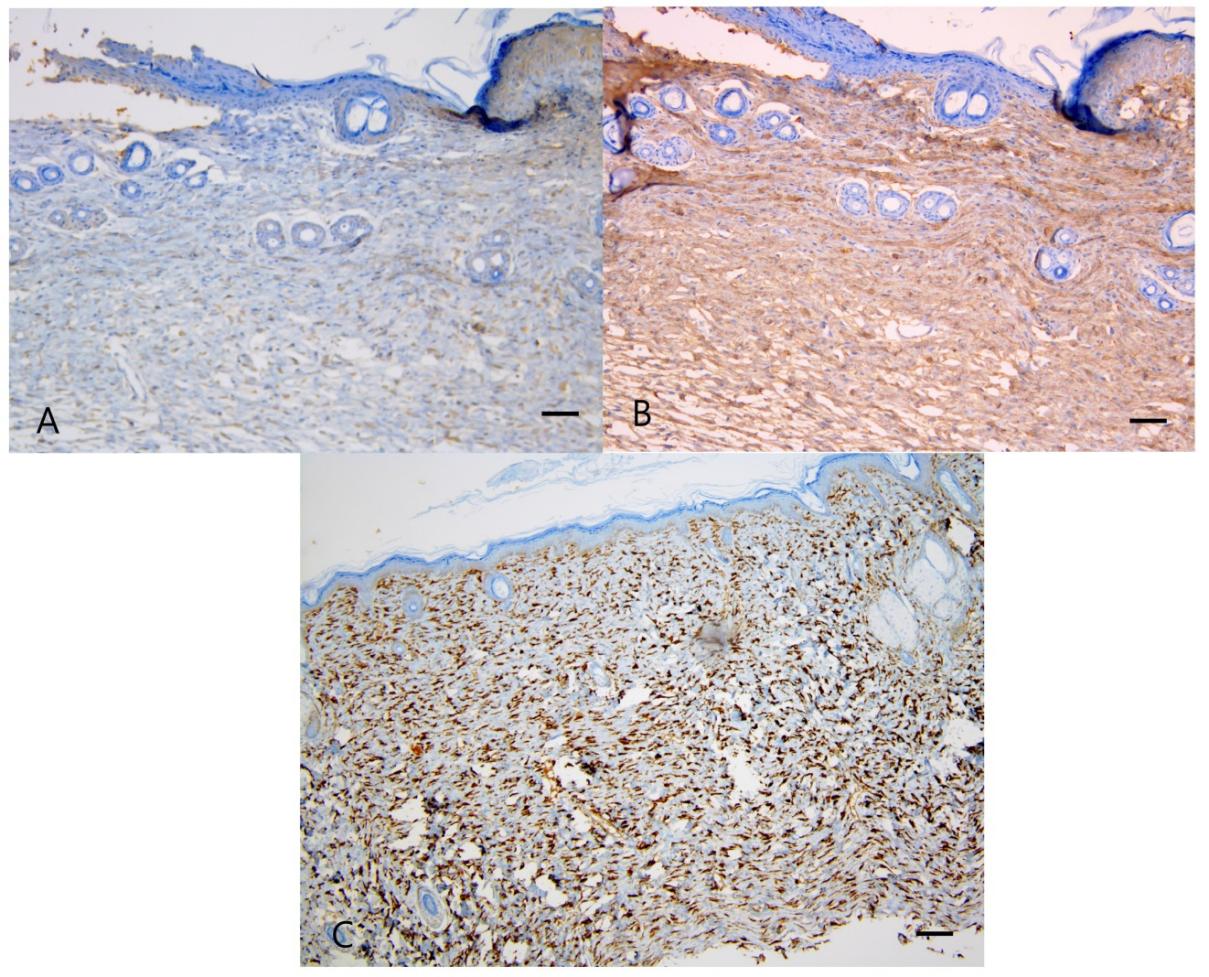
** Immunostaining for collagen type I, III, and HSP47. Captopril treatment had no significant effect on collagen type I, III, and HSP47 in normotensive and hypertensive rats.** (A) Weak collagen type I staining is seen in the control spontaneously hypertensive rat tissues, and (B) strong collagen type III staining in the control spontaneously hypertensive rat tissues (100×). (C) Intense staining of HSP47 is seen in the control spontaneously hypertensive rat tissues (100×). Scale bars in all images are 50 μm.

The expression of collagen types I and III also did not significantly differ among the groups (*P* = 0.227, 0.159, respectively), as shown in Figure 5A, 5B and Table 2. Similarly, the HSP47 expression did not significantly differ among the groups (*P* = 0.746), as shown in Figure 5C and Table 2.

## Discussion

This study was conducted to assess scar development and the effect of the ACE inhibitor, captopril, on pathological scars in genetically normotensive and hypertensive rat models. Hypertension affects the profibrotic functional changes in endothelial cells, myofibroblasts, dermal fibroblasts, and mast cells, which form keloid and hypertrophic scars. These functional changes are mediated by inflammation-induced tissue hypoxia and the renin-angiotensin system (RAS) 1. Recent research has demonstrated that a local RAS is present within healthy skin and plays a vital role in wound repair and tissue reconstruction through Ang II 14, 15, which stimulates proliferation, migration, and matrix production of human dermal fibroblasts 16, 17, as well as in keloid pathogenesis via the activation of angiotensin type 1 receptor (AT1)-dependent signaling pathway 18.

Hypertension-induced endothelial dysfunction promotes cutaneous fibrosis 19. For example, in hypertensive heart disease, elevated blood pressure causes activation of the RAS, which is followed by transforming growth factor-β (TGF-β)/Smad3 signaling activation and increased local inflammation, ECM production, and fibrosis 1, 20. Keloid induces interstitial hypertension, thereby increasing local cellular metabolic demands, decreasing metabolic substrate levels, and ultimately leading to tissue hypoxia 21, 22. This inflammation-induced hypoxic state in keloid may be caused by endothelial hypertrophy, which induces partial occlusion of microvessels. Therefore, endothelial dysfunction caused by vascular hypertension may promote hypoxia-induced fibrosis in keloids 23-25.

Several recent reports described the effect of the pharmacological antagonism of Ang II by an ACE inhibitor or AT1 antagonist, which are widely used as antihypertensive drugs, in suppressing skin wound repair, hypertrophic scars, or keloid 15, 26. Nevertheless, few studies have demonstrated the relationship between scar development and hypertension in an animal model. In the present study, we used captopril among the various ACE inhibitors because its scar reducing effect has been determined in previous in vivo studies, and the effect of lowering blood pressure in SHR has also been investigated previously 4, 9, 27. We determined the relationship between the systemic effect (lowering blood pressure) and topical effect (cutaneous scar reduction). Captopril treatment was initiated after the termination of wound healing to exclude its effect on the inflammatory phase during wound healing.

In this study, the scar area, fibroblast count, and capillary count of SHR-T were lower than that of SHR-C in hypertensive rats, while in normotensive rats only the capillary count was significantly lower in WKY-T than in WKY-C. These results suggest that the effect of the ACE inhibitor on scar development was more prominent in the hypertensive rat group than in the normotensive rat group. ACE inhibitor treatment significantly lowered the systolic BP in both SHR and WKY. In hypertensive conditions, however, elevated circulatory Ang II levels might be related to the elevation of local Ang II in SHR.

Next, examining the difference between the control groups of normotensive and hypertensive rats, the scar area and fibroblast count were found to be significantly lower in WKY-C than in SHR-C, indicating that hypertension might be related to the cutaneous scar development in this rat model, which is consistent with other reports 1. Dermal fibroblasts are mechanosensitive cells that actively interact with their microenvironment, expressed, for example, under increased fluid pressure and shear stress on the cells in the vascular lumen or walls due to hypertension 1. In this study, the fibroblast count of hypertensive rats was higher than that of normotensive rats. Further studies should examine whether there is a correlation between circulating Ang II levels and the local Ang II type 1 receptor in the scar tissue in SHR.

The immunohistochemical analysis in this study showed that VEGF expression was more prominent in SHR-C than in SHR-T. VEGF is a vascular permeability factor that promotes neovascularization and cell growth 28, 29. In addition to being a potent endothelial cell-specific cytokine, it is also a mitogenic, chemotactic factor in vitro and an inducer of angiogenesis in vivo 30-32. Ang II accelerates neovascularization and promotes the release of VEGF and platelet-derived growth factor 15, 33. The decrease in VEGF expression in SHR-T observed in this study may thus be related to the downregulation of Ang II in SHR-T. HSP47, a 47-kDa heat shock protein, is a collagen-specific molecular chaperone localized in the endoplasmic reticulum that plays an essential role in collagen biosynthesis in skin fibrosis 34. In this study, the expression of HSP47 and collagen type I and III did not differ significantly among the groups, in contrast to the histopathology results. Protein non-specificity for rat wounds and our semi-quantitative measurement method are among the limitations of this study.

α-SMA is a marker for myofibroblasts and is detected in fibroblasts and vascular endothelial cells 35. Myofibroblasts play a crucial role in wound contraction and are involved in the remodeling of granulation tissue. However, in cases of excessive scarring and fibrosis, myofibroblasts persist and contribute to excessive collagen production. In a mouse burn model, the level of α-SMA expression was shown to correlate with the severity of scarring 36. In this study, α-SMA expression was more prominent in SHR-C and WKY-C than in SHR-T and WKY-T, respectively. The Ki67 proliferation index was higher in SHR-C than in SHR-T. Ki67 is a marker for epidermal proliferation 13, and the number of Ki67-positive proliferating keratinocytes was significantly decreased in SHR-T compared to SHR-C. This phenomenon could be related to the inhibition of keratinocyte migration inhibited by angiotensin II receptor blockade 17.

This study had several limitations. First, anesthesia might have affected the plasma renin activity and circulatory Ang II levels in the rats. However, any effect of anesthesia should have disappeared, because we started the captopril treatment 3 weeks after the creation of the wound under anesthesia. Second, the wound healing process in rats is different from that in humans; rats have loose skin and subcutaneous panniculus carnosus; therefore, wound healing occurs via contraction and collagen formation 37. Consequently, the scars that developed in the rats might not have been pathological. However, the differences in the scar development between normotensive and hypertensive rats and between captopril-treated and control hypertensive rats were significant. We believe that this finding is relevant, although the issue of a "pathological scar" is unresolved. Third, we measured the blood pressure of spontaneously hypertensive and normotensive rats using a tail-cuff system because of its non-invasiveness, according to the previous studies on rat experiments 38-39. However, the blood pressure measurement of rats using the telemetry system is a more precise and better method than the tail-cuff system.

In this study, large scars tended to develop more often in hypertensive rats than in normotensive rats. The ACE inhibitor significantly reduced the scars in the hypertensive rats, while it did not reduce the scars effectively in the normotensive rats. Based on these results, blood pressure might have affected scar development in the hypertensive rat wound model used in this study. Moreover, the ACE inhibitor would be effective at reducing scars that develop only in hypertensive conditions. Further studies are necessary to demonstrate the mechanisms of action of ACE inhibitors on pathological scars in an established animal scar model using multiple parameters, such as plasma Ang II levels and tissue AT1 receptor levels.

## Figures and Tables

**Table 1:** Summary of the histopathological data.

	WKY-C	WKY-T	SHR-C	SHR-T	*P*-value	Post hoc
Scar area (× 10^3^ µm)	1.9 ± 0.4	1.3 ± 0.5	5±1.2	1.8±0.6	< 0.0001	1=2=4<3
Fibroblast count	91.5±19.1	67.1±20.6	212.1±51.1	64.8±20.1	< 0.0001	1=2=4<3
Capillary count	27.9±8.7	11.5±9	25.6±10.3	12.1±5.4	0.0004	2=4<1=3

**Abbreviations:** WKY-C, control Wistar Kyoto rats; WKY-T, captopril-treated Wistar Kyoto rats; SHR-C, control spontaneously hypertensive rats; SHR-T, captopril-treated spontaneously hypertensive rats. All data are expressed as the mean ± SD. Statistical significance at *P* < 0.05 based on an ANOVA followed by a *post hoc* Bonferroni correction for multiple comparisons.

**Table 2:** Summary of the immunohistochemical data.

	WKY-C	WKY-T	SHR-C	SHR-T	*P*-value	Post hoc
HSP47 expression score	2.6±0.5	2.5±0.5	2.7±0.5	2.6±0.5	0.746	
Collagen type I expression score	1.1±0.5	0.9±0.7	1.1±0.7	0.7±0.7	0.227	
Collagen type III expression score	2.6±0.5	2.4±0.5	2.8±0.4	2.4±0.5	0.159	
α-SMA expression score	2.3±0.5	1.5±0.5	2.7±0.5	1.3±0.5	0.0000	2=4<1=3
Ki67 proliferation index	2.4±1.7	1.1±0.5	5.6±2.4	1.2±0.5	0.0000	1=2=4<3
VEGF expression score	3.1±1.1	2.6±1.0	3.7±1.3	1.6±1.3	0.0000	2=4<1=3

**Abbreviations:** WKY-C, control Wistar Kyoto rats; WKY-T, captopril-treated Wistar Kyoto rats; SHR-C, control spontaneously hypertensive rats; SHR-T, captopril-treated spontaneously hypertensive rats. All data are expressed as the mean ± SD. Statistical significance at *P* < 0.05 based on Kruskal-Wallis test followed by *post hoc* Bonferroni correction for multiple comparisons.

## Abbreviations

ECM: extracellular matrix
ACE: angiotensin-converting enzyme
WKY: Wistar Kyoto rats
SHR: spontaneously hypertensive rats
H&E: hematoxylin and eosin
FFPE: formalin-fixed paraffin-embedded
ANOVA: one-way analysis of variance
RAS: renin-angiotensin system
Ang II: angiotensin II
AT1: angiotensin type 1 receptor
VEGF: vascular endothelial growth factor
BP: blood pressure
